# Growth Differentiation Factor-15 Suppresses Maturation and Function of Dendritic Cells and Inhibits Tumor-Specific Immune Response

**DOI:** 10.1371/journal.pone.0078618

**Published:** 2013-11-13

**Authors:** Zhizhong Zhou, Weina Li, Yang Song, Lili Wang, Kuo Zhang, Jing Yang, Wei Zhang, Haichuan Su, Yingqi Zhang

**Affiliations:** 1 The State Key Laboratory of Cancer Biology, Fourth Military Medical University, Xi'an, Shaanxi, China; 2 Department of Biopharmaceutics, Fourth Military Medical University, Xi'an, Shaanxi, China; 3 Department of Oncology, Tangdu Hospital, Xi'an, Shaanxi, China; 4 School of Life Sciences, Northwest University, Xi'an, Shaanxi, China; Istituto Superiore di Sanità, Italy

## Abstract

Dendritic cells (DCs) play a key role in the initiation stage of an antigen-specific immune response. A variety of tumor-derived factors (TDFs) can suppress DC maturation and function, resulting in defects in the tumor-specific immune response. To identify unknown TDFs that may suppress DCs maturation and function, we established a high-throughput screening technology based on a human liver tumor T7 phage cDNA library and screened all of the proteins derived from hepatoma cells that potentially interact with immature DCs. Growth/differentiation factor-15 (GDF-15) was detected and chosen for further study. By incubation of DCs cultures with GDF-15, we demonstrate that GDF-15 can inhibit surface protrusion formation during DC maturation; suppress the membrane expression of CD83, CD86 and HLA-DR on DCs; enhance phagocytosis by DCs; reduce IL-12 and elevate TGF-β1 secretion by DCs; inhibit T cell stimulation and cytotoxic T lymphocyte (CTL) activation by DCs. By building tumor-bearing mouse models, we demonstrate that GDF-15 can inhibit the ability of DCs to stimulate a tumor-specific immune response *in vivo*. These results indicate that GDF-15 may be one of the critical molecules that inhibit DC maturation and function and are involved in tumor immune escape. Thus, GDF-15 may be a novel target in tumor immunotherapy.

## Introduction

Dendritic cells (DCs), known as the most powerful professional antigen-presenting cells (APCs), initiate an antigen-specific immune response by the recognition, acquisition, processing and presentation of antigens to naïve resting T cells [1]. Unlike mature DCs (mDCs), iDCs can induce immune dysfunction and immune tolerance by various mechanisms, partly facilitating tumor escape [2]. Defects in the DC system are one of the main factors responsible for tumor immune escape. A variety of malignant cancers can recruit immature DCs (iDCs) to the tumor site and impede the cells' differentiation into functional APCs via the influence of tumor-derived factors (TDFs) that are enriched in the tumor microenvironment [1]. Besides the known tumor-derived immunosuppressive factors such as IL-6 and VEGF [3], there are still many unknown TDFs needed to be identified and studied.

Phage display has been an important tool for both basic research and drug discovery. Phage display technology allows small peptides and protein libraries to be presented on the surface of filamentous phages and permits selection of specific peptides and proteins with high affinity. This technology has been used to identify peptides for species-specific recognition of peptides. Biopanning of phage-displayed peptide libraries on intact cells in culture and on the tissues has proven successful for isolating peptides that show high cell and tissue specificities. This approach may be used to distinguish cancer cells from normal cells as well as to enable selective binding to different tumor types, even those with similar classifications [4].

To identify unknown TDFs that may suppress DC maturation and function, we established a high-throughput screening technology based on a human liver tumor T7 phage cDNA library and screened all of the proteins derived from hepatoma cells that potentially interact with iDCs. Among the tumor-related proteins that we screened, GDF-15 was chosen for further study according to the homologous alignment analysis and cell-based ELISA results.

Growth/differentiation factor-15 (GDF-15), also known as macrophage inhibitory cytokine-1 (MIC-1), prostate-derived factor (PDF), placental TGF-β (PTGF-β), placental bone morphogenetic protein (PLAB), and non-steroidal anti-inflammatory drug-activated gene-1 (NAG-1), is a member of the transforming growth factor-β (TGF-β) superfamily. The other members of GDF (such as GDF-1, GDF-3, GDF-5, GDF-7 and GDF-10) are mainly regulators of cell growth and differentiation in both embryonic and adult tissues, even play a role in skeletal morphogenesis. GDF-15 also plays a key role in prenatal development [5], [6] and the regulation of both cellular responses to stress signals and inflammation and tissue repair after acute injuries in adulthood. The dysregulation of GDF-15 expression and signaling pathways has been associated with diverse human diseases and cancer progression. In addition, little is known about the interaction between GDF-15 and immune cells in the tumor microenvironment.

Our research investigated the effects of GDF-15 on the maturation and function of DCs and reveals the possible role of GDF-15 in tumor immune escape.

## Materials and Methods

### Ethics statement

All healthy donors were confirmed to have given written informed consent to a tissue and blood procurement study allowing *ex vivo* experimentation, which is approved by Xijing Hospital. Animal research was carried out in strict accordance with the recommendations in the Guide for the Care and Use of Laboratory Animals of the National Institutes of Health in China. The protocol was approved by the Committee on the Ethics of Animal Experiments of Xijing Hospital (Permit Number: XJYYLL-2013608).

### Generation of human peripheral blood monocyte (PBMC)-derived DCs

Xijing Hospital approval was received for the studies, and the informed consent of all of the participating subjects was obtained. Human PBMCs from normal healthy donors were isolated using Ficoll density gradient centrifugation. CD14^+^ cells were positively selected using human CD14 MicroBeads (MiltenyiBiotec, Germany), according to the manufacturer's instructions. The mean purity of the obtained CD14^+^ cells was greater than 95%, as revealed by flow cytometry. The CD14^+^ cells were subsequently cultured in 12-well plates (5×10^5^/mL) in complete RPMI 1640 medium supplemented with 10% heat-inactivated FBS (HyClone, USA), 50 ng/mL rhGM-CSF, 10 ng/mL rhIL-4 (PeproTech, USA). The cells were fed with fresh medium (half of the original medium volume) containing 50 ng/mL rhGM-CSF and 10 ng/mL rhIL-4 on days 2, 4 and 6. mDCs were obtained from iDCs by cultivation for six days, as described above, followed by stimulation with 25 ng/mL rhTNF-α (PeproTech, USA) for an additional 48 h. For control purposes, iDCs remained in culture for another 48 h in rhIL-4/rhGM-CSF medium without addition of rhTNF-α. All cells were harvested on day 8 for further experiments and analysis.

### Phage display

*In vitro* screening procedures were performed as described in the instruction manual of the kit. Briefly, the CD14^+^ cells were washed and incubated with the T7 phage peptide library of human liver tumor cDNA (Novagen, USA) for 30 min. The unbound phages were amplified for the subsequent rounds of screening. Then iDCs were washed twice with PBS and cultured with serum-free medium containing 2% BSA for 2 h to clear the surface receptors. Next, the cells were incubated for 30 min after the addition of the T7 phage peptide library of human liver tumor cDNA (Novagen, USA). After the incubation, the cells were pelleted by centrifugation at 1500 rpm for 2 min. After cells were washed twice with Tris-buffer saline solution (TBS), resuspended in elution buffer and centrifuged, the supernatant was collected, and the iDCs were removed by centrifugation. The T7 phage in the supernatant was then amplified in BLT5403 bacteria (Novagen, USA). This screening process was repeated four times before culturing the T7 phage on an LB-agarose plate. The phages recovered from the last round of the screening were cloned and amplified for the cell-based ELISA screening. Briefly, the iDCs (CD14^+^ cells and mDCs were used as negative controls) were blocked with 3% nonfat milk. Then, the cells were incubated with the amplified T7 phage in 96-well plates at 37°C for 1 h. Next, a T7 Tail Fiber monoclonal antibody (Novagen, USA) was added, followed by another incubation at 37°C for 1 h. Finally, HRP-conjugated sheep anti-mouse polyclonal antibodies were added. Thirty minutes later, colorimetric detection was performed and the OD_450nm_ was recorded using a spectrophotometer. Positive phage clones, which bound to iDCs but not to CD14^+^ cells or mDCs, were selected for PCR. The nucleotide sequences were then assessed for homologous alignment using GenBank, and a series of related proteins were identified for further analysis.

### Incubation of DCs cultures with GDF-15

rhGDF-15 (R&D, USA) was added at the following concentrations to the cultured cells from day 0 to day 8: 5 ng/mL GDF-15, 10 ng/mL GDF-15, 20 ng/mL GDF-15, 50 ng/mL GDF-15. In addition, 5 ng/mL TGF-β (PeproTech, USA) and 20 ng/mL IL-10 (ABI, Canada) were separately added as positive controls. For analysis, the cells were harvested on day 8 after stimulation with 25 ng/mL rhTNF-α on day 6 for an additional 48 h of culture.

### Scanning electron microscopy

Cells grown on glass coverslips were washed twice with PBS and fixed in 3% glutaraldehyde at 4°C for 24 h. The samples were then prepared for scanning electron microscopy and investigated using an S-3400N device (HITACHI, Japan).

### Quantitative PCR

The cells were harvested, and the total mRNA was isolated using Trizol (TaKaRa) and further transcribed according to PrimeScript® RT Reagent Kit (TaKaRa, Japan) standard protocols. For real-time PCR, cDNA amplification was monitored using FastStart Universal SYBR Green Master (ROX) (Roche, USA) on the Opticon Monitor 3 System. The conditions for these PCRs were: 40 cycles of 95°C/15 sec and 60°C/1 min, using the following specific primers determined from PrimerBank: CD83 F: 5′> AAGGGGCAAAATGGTTCTTTCG <3′; CD83 R: 5′> GCACCTGTATG TCCCCGAG <3′; CD86 F: 5′> CTGCTCATCTATACACGGTTACC <3′; CD86 R: 5′> GGAAACG TCGTACAGTTCTGTG <3′; HLA-DR F: 5′> AGTCCCTGTGCTAGGATTTTTCA <3′; and HLA-DR R: 5′> ACATAAACTCGCCTGATTGGTC <3′. Quantitative values for the genes of interest were normalized using the housekeeping gene β-actin as an endogenous reference, and the primer sequences were as follows: β-actin F: 5′> AGCGAGCATCCCCCAAAGTT <3′; and β-actin R: 5′> GGGCACGAAGGCTCATCATT <3′. The fold-increase over the control was calculated using the relative quantification method of 2^−ΔΔ^Ct. All of the samples were analysed in triplicate.

### Flow cytometry analysis

Cells (5×10^5^) were harvested, washed twice with ice cold PBS containing 5% FBS and stained with antibodies at 4°C for 45 min before being analyzed on a FACS Calibur (Beckman, USA). The data were analyzed using FCS Express V3 software. To calculate the percentage of positive cells, a proportion of 2% false-positive events was accepted in the negative control samples. FITC-conjugated mouse anti-human mAbs were purchased from BD Pharmingen and used to detect the following surface Ags: CD83, CD86 and HLA-DR.

### FITC-dextran assay

Cells were harvested and resuspended in RPMI 1640 medium supplemented with 10% FBS. FITC-Dextran (Sigma, USA) was then added at a final concentration of 1 mg/mL and the cells were then incubated at either 37°C or 4°C. The average molecular weight of FITC-Dextran is 40 kDa. After 120 min, the cells were washed twice with ice-cold PBS and then analyzed on a FACS Calibur. The results were expressed as the delta mean fluorescence intensity (ΔMFI). For each sample, the background (the MFI of cells pulsed at 4°C) was subtracted from the MFI of cells incubated at 37°C. For confocal microscopy observation, after incubation with FITC-dextran at 37°C for 120 min, the cells were washed, fixed in 4% paraformaldehyde for 15 min at room temperature and stained with DAPI. The cells were then observed using an FV1000 microscope (Olympus, Japan).

### Elisa

Culture medium was collected when the cells were harvested for analysis. After centrifugation, the supernatants were assayed using an ELISA kit according to the manufacturer's instructions. The sensitivity limits of the kits for IL-12 and TGF-β1 detection were both less than 16 pg/mL. All of the samples were analysed in triplicate.

### Mixed lymphocyte reaction (MLR)

T cells were negatively selected using a human Pan T Cell Isolation Kit (MiltenyiBiotec, Germany), according to the manufacturer's instructions. The mean purity of the obtained CD3^+^ cells assessed by flow cytometry was found to be approximately 96%. To test the capacity of DCs to stimulate allogenic T cells, DCs were harvested and co-cultured at graded concentrations of 4×10^4^–5×10^3^ cells per well with 2×10^5^ allogenic T cells for 3–4 days in U-bottom 96-well plates. A standard MTT assay was then applied to determine cell proliferation. The optical density of the samples was analyzed at 490 nm using a microplate reader.

### Cytotoxic T lymphocyte (CTL) assay

SW480 cells were purchased from the Type Culture Collection of the Chinese Academy of Sciences, Shanghai, China. iDCs were cultured with SW480 cell lysate at a ratio of 1∶2 on day 6 and stimulated with 25 ng/mL rhTNF-α for an additional 48 h. The resultant mDCs (4×10^4^) were then co-cultured with T cells (2×10^5^) for 3–4 days in U-bottom 96-well plates. The culture supernatants were discarded, and the SW480 tumor cells, used as target cells, were added to the wells containing the T cells (effector cells) at different target-to-effector ratios (1∶10, 1∶20, 1∶40 or 1∶80). After co-culturing for 36 h, the supernatants were collected for the examination of cell death using a colorimetric lactate dehydrogenase (LDH) assay kit (Cayman, USA) according to the manufacturer's manual.

### Generation and identification of murine bone marrow-derived DCs (BMDCs)

Mice were purchased from center of animal research in Fourth Military Medical University. Bone marrow was flushed from the tibias and femurs of 8- to 10-week-old male BALB/c mice and depleted of red blood cells using Red Blood Cell Lysing Buffer (Solarbio, China). The cells were plated in six-well culture plates (10^6^cells/mL; 2 mL/well) in RPMI-1640 supplemented with 10% FBS, 20 ng/mL rmGM-CSF and 10 ng/mL rmIL-4 at 37°C in 5% CO_2_. On day 2 of the culture, floating cells were gently removed, and fresh medium was added. On days 4 and 6, the cells were fed with fresh medium (half of the original medium volume). On day 6, murine mDCs were obtained by stimulation with CT26 cells lysate at a ratio of 1∶2 for an additional 48 h and murine iDCs remained in culture for another 48 h in rmIL-4/rmGM-CSF medium without addition of CT26 cells lysate. All cells were harvested on day 8 for further experiments and analysis. CT26 cells were purchased from the Type Culture Collection of the Chinese Academy of Sciences, Shanghai, China. GDF-15-treated DCs received rhGDF-15 at a concentration of 50 ng/mL during the culture. FITC-conjugated anti-mouse H-2Db (eBioscience, USA), PE-conjugated mouse anti-mouse I-A [b] (BD Pharmingen, USA) and FITC-conjugated anti-mouse CD80 (eBioscience, USA) mAbs were then used to detect surface Ags on the murine DCs.

### Tumor-bearing mouse models

For the *in vivo* experiments, 10- to 12-week-old male BALB/c mice of 18–22 g in body weight were randomly divided into four groups, with five mice in each group. In the control group, CT26 tumor cells (2×10^6^) were dorsally subcutaneously injected into BALB/c mice without murine DCs. In the other groups, CT26 tumor cells (2×10^6^) were co-injected with murine iDCs, GDF-15-treated DCs or mDCs (1×10^6^) in 200 μL PBS. Two weeks after tumor challenge, the tumor growth was monitored by measuring the tumor length (L) and width (W) with a sliding caliper. The tumor size was then calculated using the equation V = 0.5×L×W^2^. Four weeks after inoculation, the tumor mass was excised and the tumor weight was measured.

### Statistical analysis

A statistical analysis was performed using GraphPad Prism software version 5 (GraphPad Software, San Diego, CA, USA). The results were expressed as the means ± SD. Mann-Whitney U-test was used for statistical analysis. Statistical analyses refer to comparisons between mDCs and iDCs or GDF-15-treated DCs. P<0.05 was considered statistically significant.

## Results

### Specific enrichment of iDCs-bound phages

**Figure 1:**
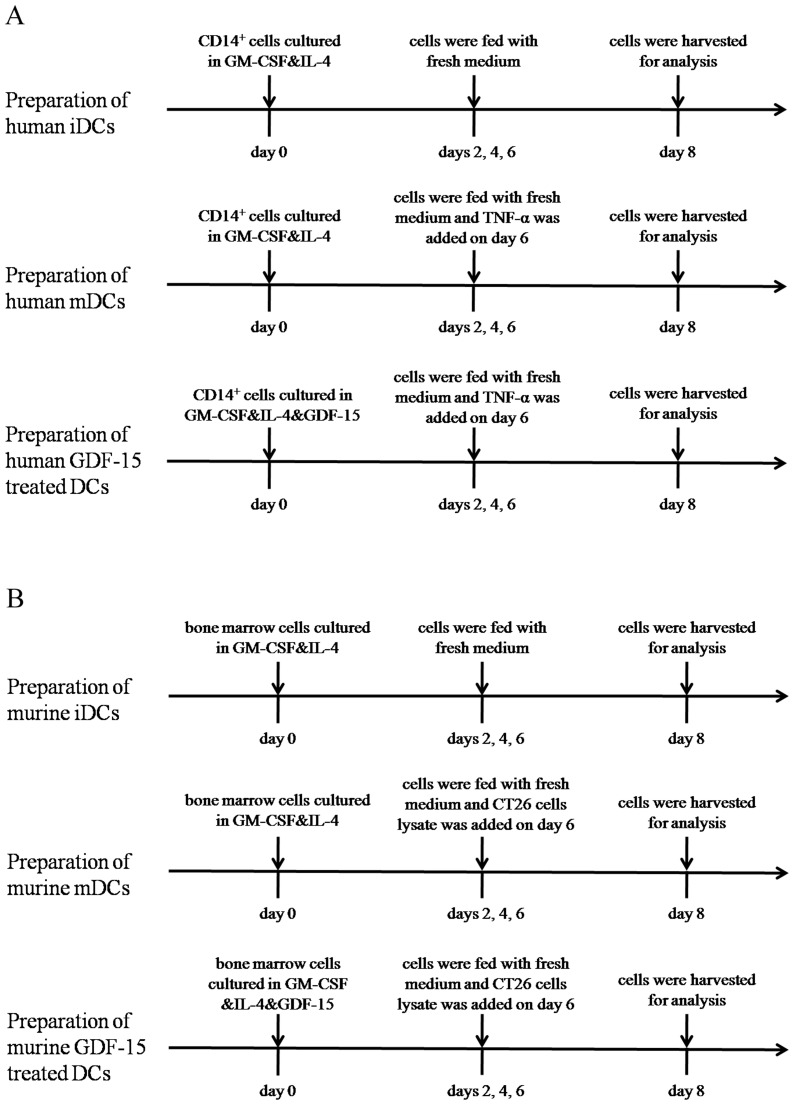
Preparation and treatment time lines for human and murine DCs. (A) Human iDCs were obtained from CD14^+^ cells cultured in GM-CSF and IL-4 for 8 days. Human mDCs were obtained from CD14^+^ cells stimulated with GM-CSF and IL-4 for 8 days and stimulated by TNF-α in the last 2 days. Human GDF-15 treated DCs were obtained from CD14^+^ cells cultured in GM-CSF, IL-4 and GDF-15 for 8 days and stimulated by TNF-α in the last 2 days. (B) Murine iDCs were obtained from bone marrow cells cultured in GM-CSF and IL-4 for 8 days. Murine mDCs were obtained from bone marrow cells cultured in GM-CSF and IL-4 for 8 days and stimulated by CT26 cells lysate in the last 2 days. Murine GDF-15 treated DCs were obtained from bone marrow cells cultured in GM-CSF, IL-4 and GDF-15 for 8 days and stimulated by CT26 cells lysate in the last 2 days.

**Figure 2:**
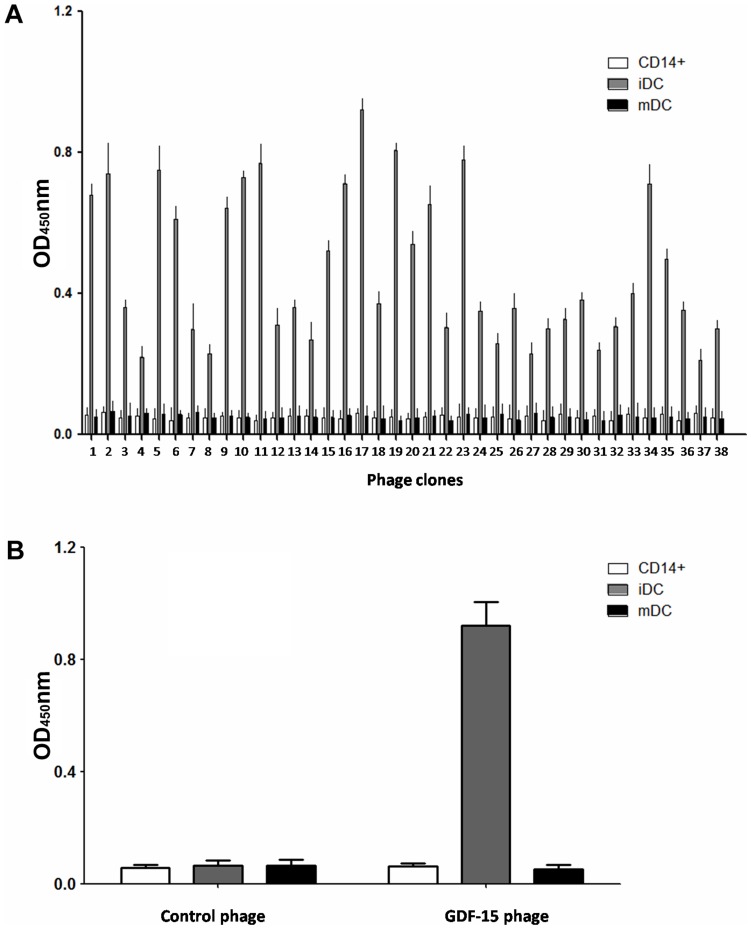
Confirmation of *in vitro* binding by cell-based ELISA. (A) Identification of the binding selectivity of the 38 clones by cell-based ELISA. The iDCs (CD14^+^ cells and mDCs were used as negative controls) were incubated with the amplified T7 phage in 96-well plates at 37°C for 1 h. Phage clones binding to cells were detected by the T7 Tail Fiber monoclonal antibody and following sheep anti-mouse IgG conjugated with HRP. (B) Specificity of the GDF-15-expressing phage to iDCs. Control phage: uncombined phage in the first round of panning. All assays were carried out in triplicate and the error bars indicate standard deviation.

We isolated CD14^+^ cells from the PBMCs of healthy donors and then stimulated the CD14^+^ cells with rhGM-CSF and rhIL-4 to generate iDCs. Finally, we stimulated the iDCs with rhTNF-α to generate mDCs (Fig. 1A). Phages that specifically bound to iDCs were identified through four rounds of *in vitro* selection. In each round, the bound phages were rescued and amplified in BLT5403 bacteria for the subsequent panning, whereas the unbound phages were removed by washing with TBS. After four rounds of biopanning, the cell-based ELISA results of selected phages were shown in Figure 2A. The output/input ratio of phages after each round of biopanning used to determine the enrichment was shown in Table 1. These results indicated that phages that were capable of specifically binding to iDCs were enriched.

**Table 1:** Enrichment of the phages in each round of biopanning.

Rounds	Input phages (pfu/mL)	Output phages (pfu/mL)	Ratio (Output/Input)
1	6.2×10^7^	3.8×10^5^	6.13×10^−3^
2	6.2×10^7^	4.2×10^5^	6.77×10^−3^
3	6.2×10^7^	6.3×10^5^	1.02×10^−2^
4	6.2×10^7^	8.4×10^5^	1.35×10^−2^

### Confirmation of *in vitro* binding by cell-based ELISA

To identify the affinity of the selected phages with iDCs, cell-based ELISA was performed to exclude false-positive colonies and those binding with equal affinity to CD14^+^ cells and mDCs. Results indicated that these phage clones could bind effectively to iDCs compared with CD14^+^ cells and mDCs. Figure 2B indicated that these phage clones could bind effectively to iDCs compared with CD14^+^ cells and mDCs.

### Homologous alignment analysis

Before screening TDFs that bound to iDCs, we pre-incubated a T7 phage peptide library with CD14^+^ cells and obtained a T7 phage in the supernatant. After four rounds of screening, positive phage clones were amplified by PCR and sequenced. The encoded amino acid sequences were determined, and 65 positive phage oligopeptides mainly included 38 sequences. The BLAST result showed that these hepatoma-related membrane molecules and homologous secreted proteins bound to iDCs from the T7 phage peptide library of human liver tumor cDNA (Table 2). As GDF-15 sequence repeated most frequently in the 65 screened sequences and cell-based ELISA results also showed that GDF-15 had the highest binding affinity than other proteins, the effects of GDF-15 on the maturation and function of DCs were then further studied.

**Table 2:** The BLAST results of the 38 screened hepatoma-related homologous proteins.

Num	Gene	Abbreviation	mRNA Gene ID
1	Alpha-fetoprotein precursor	AFP	NM_001134
2	Alanine-glyoxylate aminotransferase	AGXT	NM_000030
3	Albumin	ALB	NM_000477
4	AlkB, alkylation repair homolog 7 (E. coli)	ALKBH7	NM_032306
5	Alpha-1-microglobulin precursor	AMBP	NM_001633
6	Apolipoprotein H (beta-2-glycoprotein I)	APOH	NM_000042
7	ArfGAP with RhoGAP domain, ankyrin repeat and PH domain 1	ARAP1	NM_001040118
8	ATG2 autophagy related 2 homolog A (S. cerevisiae)	ATG2A	NM_015104
9	ATPase, Na^+^/K^+^ transporting, alpha 1 polypeptide	ATP1A1	NM_000701
10	Complement component 3	C3	NM_000064
11	Complement component 9	C9	NM_001737
12	Cyclin G1(1,2)	CCNG1	1: NM_004060q 2: NM_199246
13	Clathrin, heavy chain (Hc)	CLTC	NM_004859
14	Electron-transfer-flavoprotein, beta polypeptide	ETFB	NM_001014763
15	Ferritin, heavy polypeptide 1	FTH1	NM_002032
16	Ferritin, light polypeptide	FTL	NM_000146
17	Growth differentiation factor 15	GDF15	NM_004864
18	G protein-coupled receptor 116	GPR116	NM_001098518
19	H1 histone family, member 0	H1F0	NM_005318
20	High density lipoprotein binding protein	HDLBP	NM_005336
21	Neuropeptide W	NPW	NM_001099456
22	Pescadillo homolog 1, containing BRCT domain (zebrafish)	PES1	NM_014303
23	POM121 membrane glycoprotein C	POM121C	NM_001099415
24	P450 (cytochrome) oxidoreductase	POR	NM_000941
25	Ribosomal protein S17	RPS17	NM_001021
26	Ribosomal protein S21	RPS21	NM_001024
27	Small EDRK-rich factor 2	SERF2	NM_001018108
28	Serpin peptidase inhibitor, clade H (heat shock protein 47), member 1, (collagen binding protein 1)	SERPINH1	NM_001235
29	Solute carrier family 25(mitochondrial carrier; phosphate carrier), member 3	SLC25A3	NM_002635
30	Smg-5 homolog, nonsense mediated mRNA decayfactor (C. elegans)	SMG5	NM_015327
31	Signal sequence receptor, alpha	SSR1	NM_003144
32	Structure specific recognition protein 1	SSRP1	NM_003146
33	Threonyl-tRNA synthetase 2, mitochondrial (putative)	TARS2	NM_025150
34	Transmembrane protein 49	TMEM49	NM_030938
35	Unc-51-like kinase 1 (C. elegans)	ΜLK1	NM_003565
36	WAS protein family homolog 1	WASH1	NM_182905
37	WAS protein family homolog 2 pseudogene	WASH2P	NM_198943
38	SUMO1 activating enzyme subunit 1(a, b, c)	SAE1	A: NM_005500 b: NM_001145713 c: NM_001145714

### GDF-15 reduces protrusion formation during DC maturation

**Figure 3:**
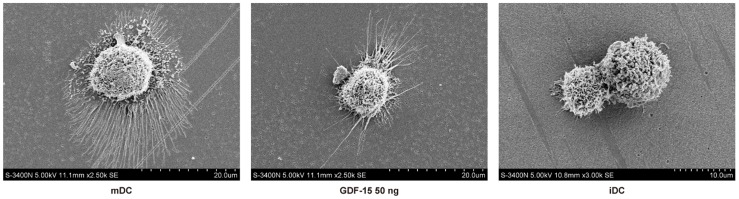
GDF-15 affects the morphologies of DCs. Scanning electron micrographs of an iDC, an mDC and a GDF-15-treated (50 ng/mL) DC. CD14+ cells were grown on glass coverslips and induced to form DCs. Samples for scanning electron microscopy were then prepared and investigated using an S-3400N microscope. Original magnification ×3000 or ×2500.

Surface protrusion is an important morphological sign of DC maturation. Scanning electron microscopy showed that surface protrusions were not obvious in the iDCs, whereas the mDCs had a typical morphology, with many long, radiating surface protrusions. GDF-15-treated DCs were more iDC-like in morphology, and protrusion retraction was observed (Fig. 3).

### GDF-15 suppresses expression of maturation and costimulatory molecules in DCs

**Figure 4:**
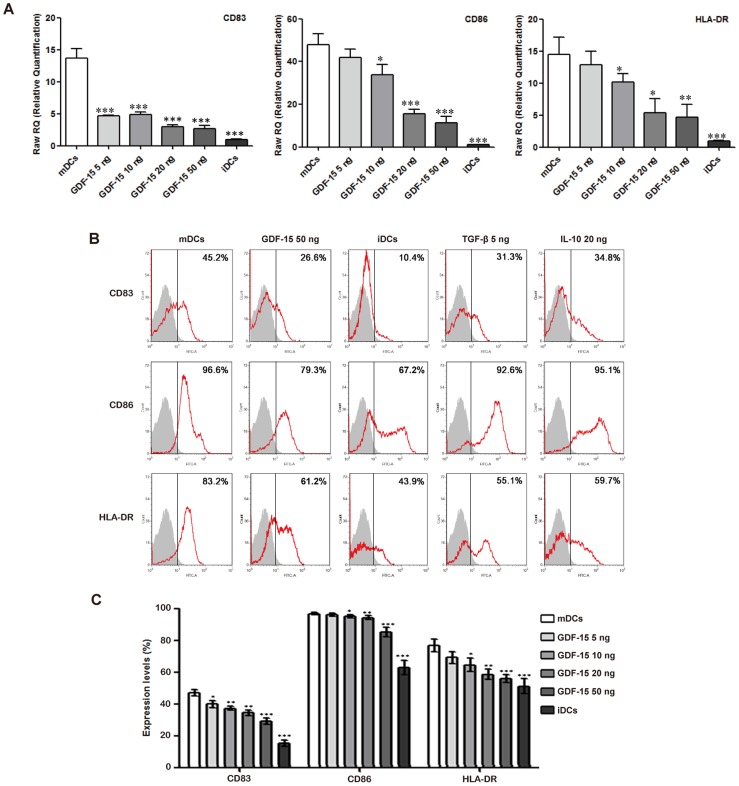
GDF-15 affects the phenotypes of DCs. (A) Quantitative PCR analysis of DC phenotypes based on the expression of CD83, CD86 and HLA-DR. The quantitative values for the genes of interest were normalized using the housekeeping gene β-actin as an endogenous reference. The fold-increase over the control was calculated using the relative quantification method of 2^−ΔΔ^Ct. Mean ± SD, n = 3. (B, C) Flow cytometry analysis of DC phenotypes based on the expression of CD83, CD86 and HLA-DR. Mean ± SD, n = 7. * P<0.05, ** P<0.01 and *** P<0.001 compared with the mDCs. The experiments were conducted in triplicate.

For the detection of surface marker expression, quantitative PCR and flow cytometry analysis were performed. As expected, the iDCs expressed lower levels of CD83, CD86 and HLA-DR than the mDCs. GDF-15 inhibited the expression of these molecules at both the mRNA (Fig. 4A) and the protein levels (Fig. 4B). Flow cytometry showed that TGF-β and IL-10 also decreased these molecules' expression (Fig. 4B). The downregulation effects of GDF-15 on DC surface marker expression were dose-dependent (Fig. 4C).

### GDF-15 enhances phagocytosis by DCs

**Figure 5:**
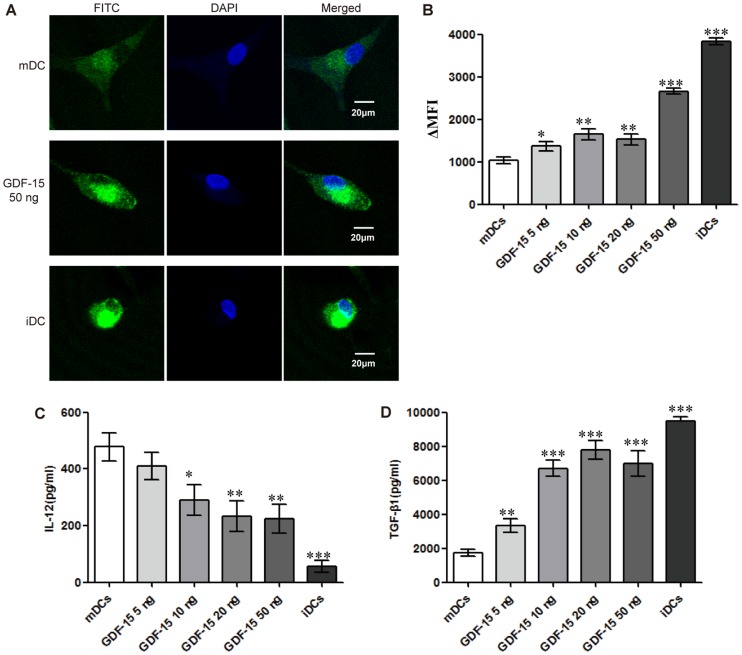
GDF-15 affects phagocytosis and cytokine secretion by DCs. (A) Confocal microscopy. CD14+ cells were cultured in a 15 mm confocal dish and induced to form DCs under different culture conditions. After incubation with FITC-dextran, fixation and staining with DAPI, the cells were observed by confocal microscopy. Original magnification ×400. The scale bars are equal to 20 μm. (B) Flow cytometry. The results are shown as the ΔMFI. For each sample, the background (MFI of the fluorescence of the cells pulsed at 4°C) was subtracted from the MFI of the cells incubated at 37°C. (C, D) Levels of cytokine secretion in DCs. The culture medium was collected when the cells were harvested for analysis. After centrifugation, IL-12 (C) and TGF-β1 (D) levels in the supernatants were detected using an ELISA kit according to the manufacturer's instructions. The OD_450nm_ was recorded using a spectrophotometer. Mean ± SD, n = 3. * P<0.05, ** P<0.01 and *** P<0.001 compared with the mDCs. The experiments were conducted in triplicate.

Phagocytosis by DCs is an important indicator in DC function evaluation that is commonly gauged by the cells' uptake of FITC-dextran. During the maturation of DCs, phagocytosis is significantly reduced. Confocal microscopy showed that the iDCs exhibited maximum intracellular FITC-dextran levels, with brightest green signal observed. Intracellular FITC-dextran also increased in GDF-15-treated DCs compared with mDCs (Fig. 5A). The ΔMFIs were determined to assess changes in phagocytosis. Flow cytometry analysis showed that the iDCs had a significantly higher ΔMFI than the mDCs. Additionally, the ΔMFI increased in the GDF-15-treated DCs in a dose-dependent manner, as expected (Fig. 5B).

### GDF-15 affects secretion of IL-12 and TGF-β1 by DCs

In general, iDCs secrete higher levels of TGF-β1 and lower levels of IL-12 then mDCs. Our results for the secretion of IL-12 and TGF-β1 from DCs were as expected. Compared with mDCs, GDF-15-treated DCs secreted a lower level of IL-12 (Fig. 5C) and a higher level of TGF-β1 (Fig. 5D), further illustrating the inhibitory effect of GDF-15 on DC maturation.

### GDF-15 inhibits T cell stimulation and CTL activation induced by DCs

**Figure 6:**
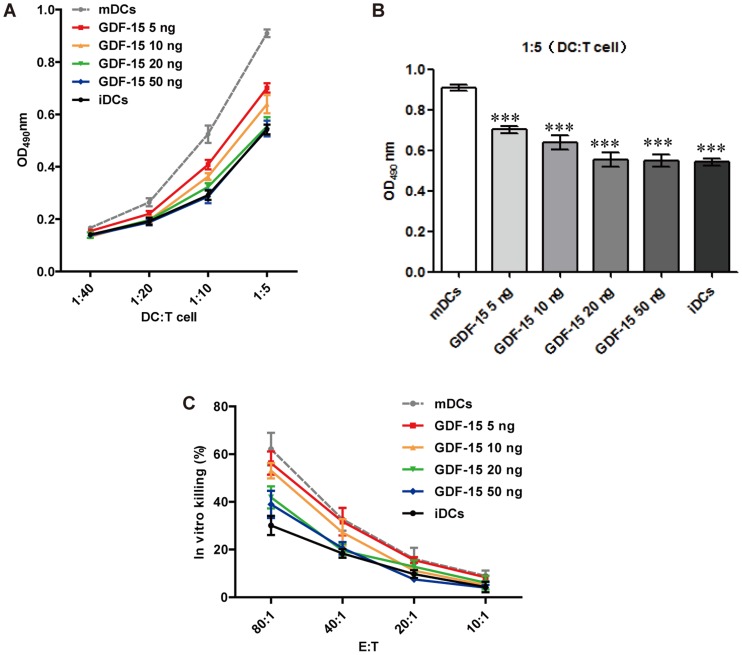
GDF-15 inhibited T cell stimulation and CTL activation induced by DCs. (A) MLR. DC-mediated T cell stimulation was analyzed in an MLR by incubating DCs obtained under different culture conditions with T lymphocytes at the indicated ratios. (B) At a DC: T cell ratio of 1∶5, the T cell-stimulatory capacity of DCs was significantly reduced by GDF-15. Mean ± SD, n = 4. (C) CTL assay. DCs obtained under different culture conditions were co-cultured with T cells at a ratio of 1∶5 for 3–4 days to generate effector cells. SW480 cells were then co-cultured with the activated T cells at a ratio of 1∶10, 1∶20, 1∶40 or 1∶80 for 36 h. The LDH in the supernatants was detected using a kit. Mean ± SD, n = 8. *** P<0.001 compared with the mDCs. The experiments were conducted in triplicate.

In the initial stage of T cell activation, the ability of DCs to stimulate T cell proliferation can reflect DCs' efficacy function as APCs. It is also known that the T cell-stimulatory capacity of mDCs is higher than the capacity of iDCs. This ability was determined by MLR experiments. After maturation in the presence of GDF-15, the DCs exhibited a reduced T cell-stimulatory capacity that was GDF-15 dose-dependent (Fig. 6A). At a DC: T cell ratio of 1∶5, a significant reduction in the capacity of mDCs to induce allogenic T cell proliferation was detected in all of the GDF-15-treated groups (Fig. 6B). By presenting a tumor antigen, mDCs interact with antigen-specific T cells and therefore activate CTLs to kill tumor cells. Measuring the LDH content of the cell culture supernatant, which reflects cell death, is one of the currently commonly used CTL assays [7]. Our results showed that at an effector-to-target cell ratio of 80∶1, T cells co-cultured with tumor cell lysate loaded DCs could significantly kill tumor cells, whereas GDF-15 inhibited the CTL activation induced by DCs in a dose-dependent manner (Fig. 6C).

### GDF-15 inhibits ability of DCs to stimulate tumor-specific immune response *in vivo*

**Figure 7:**
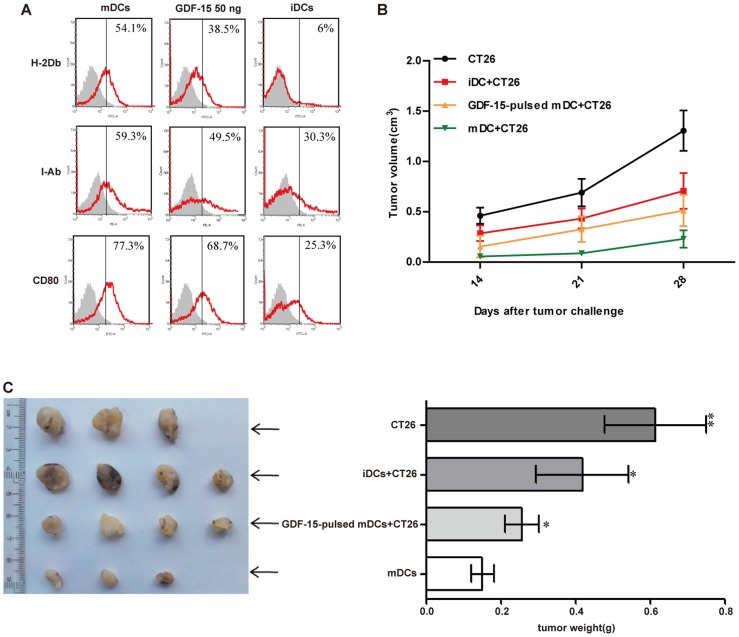
GDF-15 inhibited the ability of DCs to stimulate a tumor-specific immune response *in vivo*. (A) Flow cytometry. rhGDF-15 suppressed the expression of the murine BMDC phenotype-associated molecules H-2D^b^, I-A^b^ and CD80. (B) Tumor growth curves for different groups. CT26 tumor cells (2×10^6^) were dorsally subcutaneously injected or co-injected with murine iDCs, GDF-15-treated DCs or mature DCs (1×10^6^) into BALB/c mice (10- to 12-week-old, male). Two weeks after the tumor challenge, the tumor size was calculated. (C) Tumor weights for different groups. Each tumor was excised four weeks after inoculation, and the tumor weight was measured. Mean ± SD. * P<0.05 and ** P<0.01 compared with the mDCs.

Murine bone marrow cells were cultured *in vitro* as described (Fig. 1B). Flow cytometry analysis showed that our culture system induced murine DCs from murine bone marrow. Since there is no available commercialized murine-derived GDF-15, we tested whether human-derived GDF-15 could also work on murine DCs. The flow cytometry analysis showed that rhGDF-15 inhibited the maturation of these murine DCs and decreased surface expression of MHC I (H-2D^b^), MHC II (I-A^b^) and CD80 (Fig. 7A). Using a preparation method of DC vaccines, we loaded murine BMDCs with CT26 cells lysate and co-injected these sensitized DCs with CT26 cells into BALB/c mice. Obvious tumor masses were observed after two weeks and then measured every week. According to the tumor growth curves and tumor weights, the tumors grew fastest in the control group, whereas the mDC group exhibited the slowest tumor growth rate (Fig. 7B) and the smallest tumor size (Fig. 7C). Compared with the mDC group, GDF-15 could accelerate the tumor growth rate and increase tumor size. Additionally, CT26 cells culture medium was collected and ELISA result showed that CT26 cells did not secret GDF-15 (Fig. S3).

## Discussion

DCs play a key role in the initial stage of an antigen-specific immune response. However, iDCs can be recruited to a tumor site and prevented from maturing by various malignant cancers [8]. It is now recognized that TDFs can contribute to the dysfunction of DCs during tumor immune escape [9]. To identify new TDFs that can suppress DC maturation, we first established a high-throughput screening technology based on a human liver tumor T7 phage cDNA library. After four rounds of panning, the phage recovery rate gradually increased, and positive phage clones were effectively enriched. Then cell-based ELISA was adopted to confirm specific binding of iDCs *in vitro*. DNA sequencing and homologous alignment analysis of the selected phage clones were applied. Finally, we screened and identified these molecules derived from hepatoma cells that could potentially interact with iDCs, laying a foundation for the further study of TDF-mediated effects on DC maturation and function.

Among the identified TDFs, GDF-15 is highly expressed in various malignant cancers and is associated with the proliferation, metastasis and prognosis of colon cancer [10], ovarian cancer [11], oral squamous cell carcinoma [12], melanoma [13] and prostate cancer [14]. The GDF-15 serum level also significantly increases in diverse aggressive human cancers. Segerer [15] first reported that GDF-15 produced in decidual cells could directly induce a tolerogenic subtype of DCs, which are known to play a distinct role in the development of pro-fetal tolerance in pregnancy. Although GDF-15 is a member of the TGF-β superfamily and TGF-β has been confirmed to inhibit DC maturation and function, little is known about the interaction between GDF-15 and immune cells in the tumor microenvironment.

We first built a stable induction culture system for DCs *in vitro* according to a relevant report [16]. Then we measured the effect of GDF-15 on the viability of the cells in culture. Flow cytometry analysis showed that there were no significant differences among iDCs, mDCs and GDF-15-treated DCs (Fig. S1). We observed DC morphological changes after GDF-15 stimulation. Scanning electron microscopy showed that the morphology of GDF-15-treated DCs tended to be smooth, with the cells' protrusions retracted, resembling iDCs. In contrast, mDCs had more long surface protrusions. This observation indicated that GDF-15 may affect inner organelle development during DC maturation.

For the identification of cell phenotypes, we mainly chose the currently accepted human DC markers CD83, CD86 and HLA-DR to distinguish between iDCs and mDCs. We verified that GDF-15 could inhibit the expression of these molecules by quantitative PCR. Furthermore, flow cytometry analysis showed that the mDCs expressed relatively higher levels of CD83, CD86 and HLA-DR than the iDCs. The expression of these molecules in the mDCs was downregulated after GDF-15 stimulation in a dose-dependent manner, indicating that GDF-15 possibly affected the DCs' inhibitory function by moderating the expression of key membrane molecules. Additionally, we also treated mDCs with GDF-15 for 1 day, 2 days and 3 days, separately. However, quantitative PCR showed that GDF-15 did not suppress mDCs once they had already been activated by TNF-α (Fig. S2).

By pinocytosis and receptor-mediated endocytosis and phagocytosis, iDCs are primarily responsible for antigen capture, whereas mDCs mainly contribute to antigen presentation, as phagocytosis by DCs is gradually reduced during maturation. After incubating DCs with FITC-dextran, confocal microscopy showed that the iDCs exhibited maximum intracellular FITC-dextran levels and that FITC-dextran intake was increased in the GDF-15-treated DCs compared with the mDCs. Flow cytometry analysis also showed that the iDCs had a significantly higher ΔMFI than mDCs. The ΔMFI increased in GDF-15-treated DCs in a dose-dependent manner, as expected, indicating that GDF-15 can maintain phagocytosis by DCs and in turn suppress DC maturation.

IL-12 can stimulate the proliferation of activated T cells and induce the differentiation of naïve T cells into Th1 cells, promoting the secretion of IFN-γ and TNF-α. Li [17] found that the T cell-stimulatory capacity of IL-12-knockout DCs, generated by siRNA interference, was significantly reduced. These inductive effects of IL-12 could also be inhibited by TGF-β [18], [19]. In the current study, ELISA analysis showed that the mDCs had significantly higher IL-12 and lower TGF-β1 secretion levels than the iDCs, as expected. GDF-15 reduced IL-12 and elevated TGF-β1 production, indicating that GDF-15 suppressed DCs' ability to promote the cellular immune response.

As the most important accessory and stimulatory cells, DCs have the ability to stimulate the proliferation of T cells and to directly affect subsequent T cell killing of tumor cells. In our MLR experiments, the mDCs significantly stimulated T cell proliferation, but GDF-15 inhibited this ability. In the cellular immune response, CTLs activated by mDCs can kill tumor cells efficiently and specifically with the help of Th1and other auxiliary cells. In our CTL assay, we sensitized DCs by adding tumor cell lysate, and these sensitized mDCs effectively activated the corresponding tumor cell-killing effect of the CTLs. GDF-15 inhibited this ability of DCs to activate CTL killing. These results indicate that GDF-15 indeed inhibited the function of DCs serving as APCs that activate T cells. These DCs with functional defects may further induce T cell tolerance and dysfunction [20] or even induce the generation of regulatory T cells [21], which suppress the tumor-specific immune response.

*In vitro*, we confirmed that GDF-15 can inhibit the maturation and damage the normal function of DCs. However, whether GDF-15 can also inhibit normal function of DCs *in vivo* was still unclear. Thus, we constructed tumor-bearing mouse models based on the preparation methods used for DC vaccines to test our hypothesis. Flow cytometry was used to confirm the inhibitory effects of rhGDF-15 on murine DC maturation. To examine whether CT26 cells express GDF-15, we collected CT26 culture supernatant and applied for ELISA. Result showed that CT26 cells did not secret GDF-15 (Fig. S3). We then loaded murine BMDCs with CT26 cells lysate and co-injected these sensitized DCs with CT26 cells into BALB/c mice. We found that the resultant tumor grew fastest in the control group, whereas the mDC group had the slowest tumor growth rate and the smallest tumor size. GDF-15 accelerated the tumor growth rate and increased the tumor size. To validate the role of GDF-15 in tumor progress, we used a GDF-15 polyclonal antibody to block the GDF-15 induced maturation and the result revealed that the antibody abolished the effect of GDF-15 on inhibiting CD83, CD86 and HLA expression on mDCs *in vitro* (Fig. S4). During DCs maturation, CCR7 expression increases and is thought to be involved in DCs chemotaxis to the lymph node [22]. Thus measurement of CCR7 on murine DCs was taken by flow cytometry analysis. Results showed that the mDCs expressed higher level of CCR7 than iDCs. GDF-15 inhibited CCR7 expression on GDF-15-treated DCs (Fig. S5). This may be a possible mechanism *in vivo* that GDF-15 could interfere with migration of DCs from the site of tumor to draining LNs, leading to less tumor immunity, inhibiting the ability of DCs to activate the tumor-specific immune response *in vivo*.

Although our researches clarified the effects of GDF-15 on the maturation and function of DCs and revealed a possible role for GDF-15 during tumor immune escape, the specific mechanism for these effects has not yet been elucidated. As receptors for GDF-15 have not been identified, whether GDF-15 functions similarly to other members of the TGF-β superfamily through the Smad signaling pathway [23] or has its own independent receptors and signaling pathway remain to be further studied.

In summary, we screened and identified proteins that bind to iDCs using a T7 phage peptide library of human liver tumor cDNA. We determined that GDF-15 could inhibit DC maturation and function both *in vitro* and *in vivo*. Thus, GDF-15 may be one of the critical molecules involved in tumor immune escape and may be a novel target in tumor immunotherapy.

## Supporting Information

Figure S1 **Apoptosis analysis.** The effects of GDF-15 on the viability of the DCs in culture were measured. After 8 days of culture, iDCs, mDCs and GDF-15-treated DCs were harvested for apoptosis analysis. Viability of iDCs, mDCs and GDF-15 (50 ng/mL) treated DCs were 72.4±4.6%, 73.3±3.2% and 70.9±6.1%, respectively. Mean ± SD, n = 3. The experiments were conducted in triplicate.
(TIF)

Figure S2 **Quantitative PCR analysis of DC phenotypes based on the expression of CD83, CD86 and HLA-DR.** mDCs were treated with GDF-15 for 1 day, 2 days and 3 days, separately. The quantitative values for the genes of interest were normalized using the housekeeping gene β-actin as an endogenous reference. The fold-increase over the control was calculated using the relative quantification method of 2^−ΔΔ^Ct. Mean ± SD, n = 3. The experiments were conducted in triplicate.
(TIF)

Figure S3 **ELISA.** The OD_450nm_ was recorded using a spectrophotometer. The OD_450nm_ of CT26 cells supernatants and GDF-15 standard (500 pg/mL) are 0.006±0.004 and 0.130±0.005, respectively. Mean ± SD, n = 3.
(TIF)

Figure S4 **GDF-15 polyclonal antibody abolished the effect of GDF-15 in inhibiting CD83, CD86 and HLA expression on mDCs.** GDF-15 polyclonal antibody (20 ng/mL) was added in DCs culture. After 8 days of culture, quantitative PCR was performed. The quantitative values for the genes of interest were normalized using the housekeeping gene β-actin as an endogenous reference. The fold-increase over the control was calculated using the relative quantification method of 2^−ΔΔ^Ct. Mean ± SD, n = 3. * P<0.05, ** P<0.01 and *** P<0.001 compared with the GDF-15 (50 ng/mL) treated DCs. The experiments were conducted in triplicate.
(TIF)

Figure S5 **Measurement of CCR7 on murine DCs.** DCs were harvested on day 8 for CCR7 analysis. Flow cytometry were performed to detect CCR7 expression. CCR7 expression on iDCs, mDCs and GDF-15 (50 ng/mL) treated DCs were 21.4±2.6%, 60.3±5.2% and 43.9±3.1%, respectively. Mean ± SD, n = 3. The experiments were conducted in triplicate.
(TIF)
