# Supplementary figures and images for: Growth Differentiation Factor-15 Suppresses Maturation and Function of Dendritic Cells and Inhibits Tumor-Specific Immune Response
Source: PLoS One. 2013 Nov 13;8(11):e78618. doi: 10.1371/journal.pone.0078618 (PMC3827235; doi:10.1371/journal.pone.0078618)

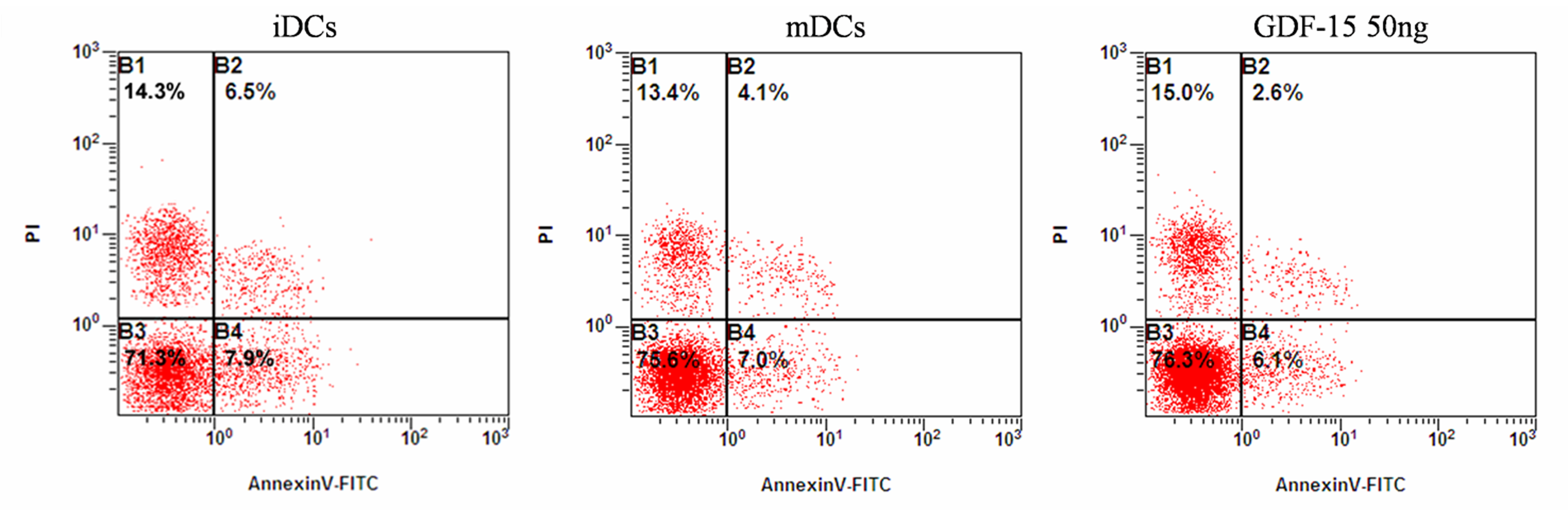

Supplement: Figure S1 — Apoptosis analysis. The effects of GDF-15 on the viability of the DCs in culture were measured. After 8 days of culture, iDCs, mDCs and GDF-15-treated DCs were harvested for apoptosis analysis. Viability of iDCs, mDCs and GDF-15 (50 ng/mL) treated DCs were 72.4±4.6%, 73.3±3.2% and 70.9±6.1%, respectively. Mean ± SD, n = 3. The experiments were conducted in triplicate. (TIF) [file pone.0078618.s001.tif]

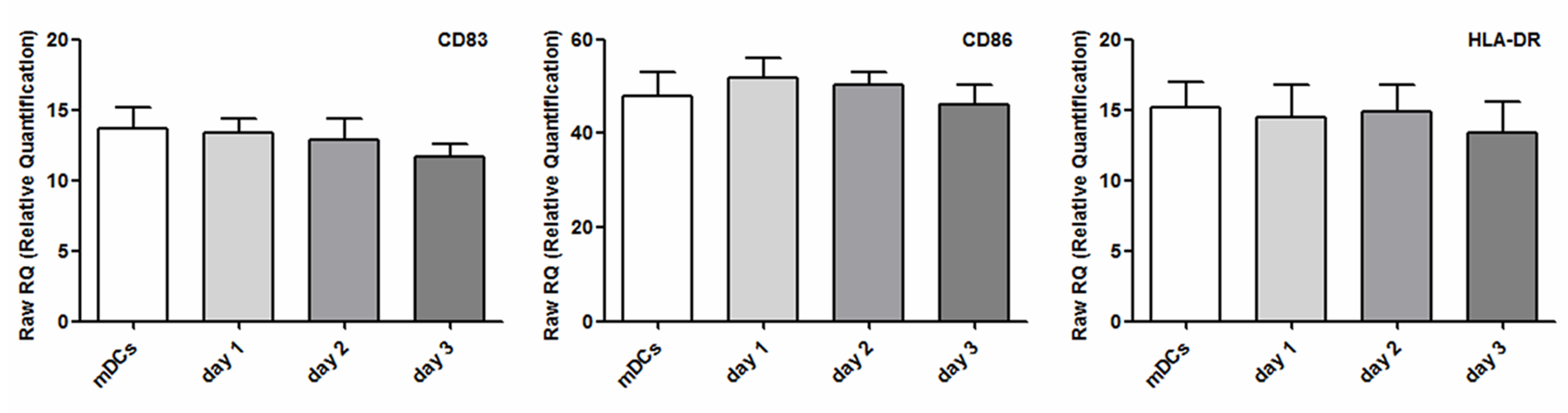

Supplement: Figure S2 — Quantitative PCR analysis of DC phenotypes based on the expression of CD83, CD86 and HLA-DR. mDCs were treated with GDF-15 for 1 day, 2 days and 3 days, separately. The quantitative values for the genes of interest were normalized using the housekeeping gene β-actin as an endogenous reference. The fold-increase over the control was calculated using the relative quantification method of 2−ΔΔCt. Mean ± SD, n = 3. The experiments were conducted in triplicate. (TIF) [file pone.0078618.s002.tif]

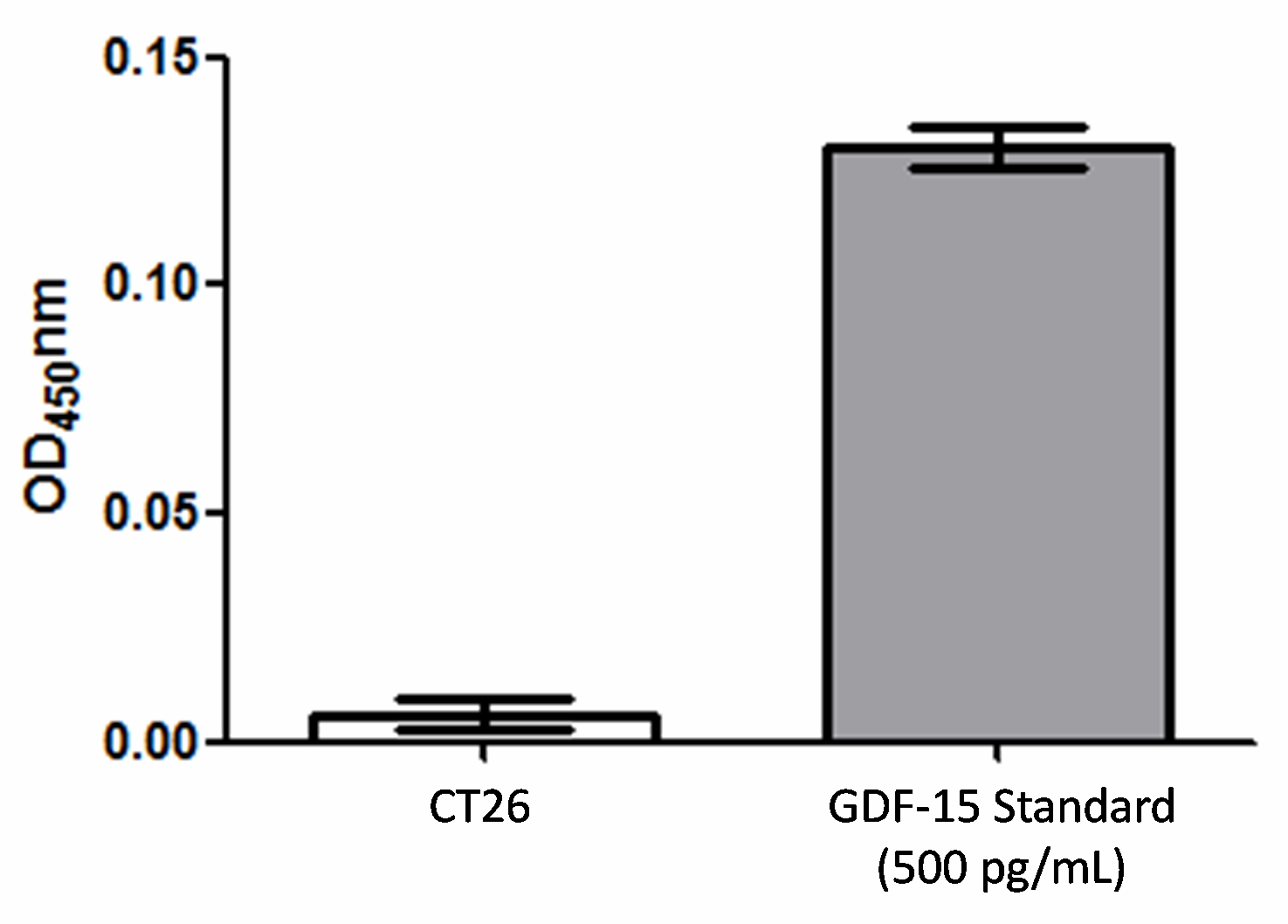

Supplement: Figure S3 — ELISA. The OD450nm was recorded using a spectrophotometer. The OD450nm of CT26 cells supernatants and GDF-15 standard (500 pg/mL) are 0.006±0.004 and 0.130±0.005, respectively. Mean ± SD, n = 3. (TIF) [file pone.0078618.s003.tif]

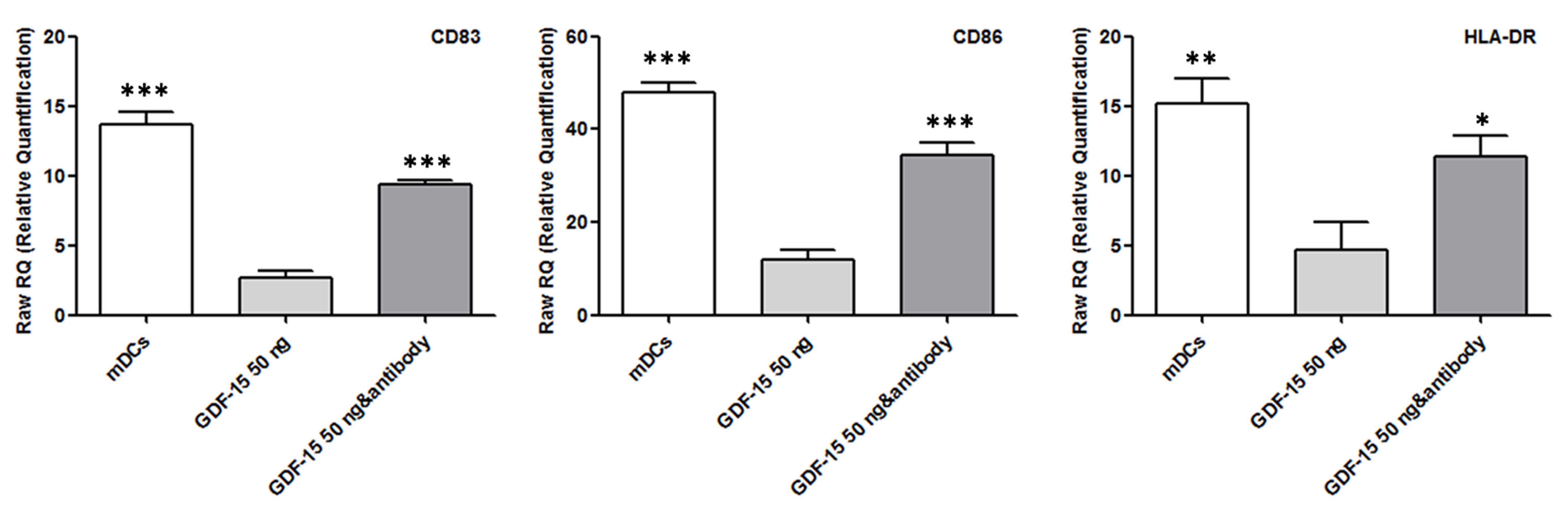

Supplement: Figure S4 — GDF-15 polyclonal antibody abolished the effect of GDF-15 in inhibiting CD83, CD86 and HLA expression on mDCs. GDF-15 polyclonal antibody (20 ng/mL) was added in DCs culture. After 8 days of culture, quantitative PCR was performed. The quantitative values for the genes of interest were normalized using the housekeeping gene β-actin as an endogenous reference. The fold-increase over the control was calculated using the relative quantification method of 2−ΔΔCt. Mean ± SD, n = 3. * P<0.05, ** P<0.01 and *** P<0.001 compared with the GDF-15 (50 ng/mL) treated DCs. The experiments were conducted in triplicate. (TIF) [file pone.0078618.s004.tif]

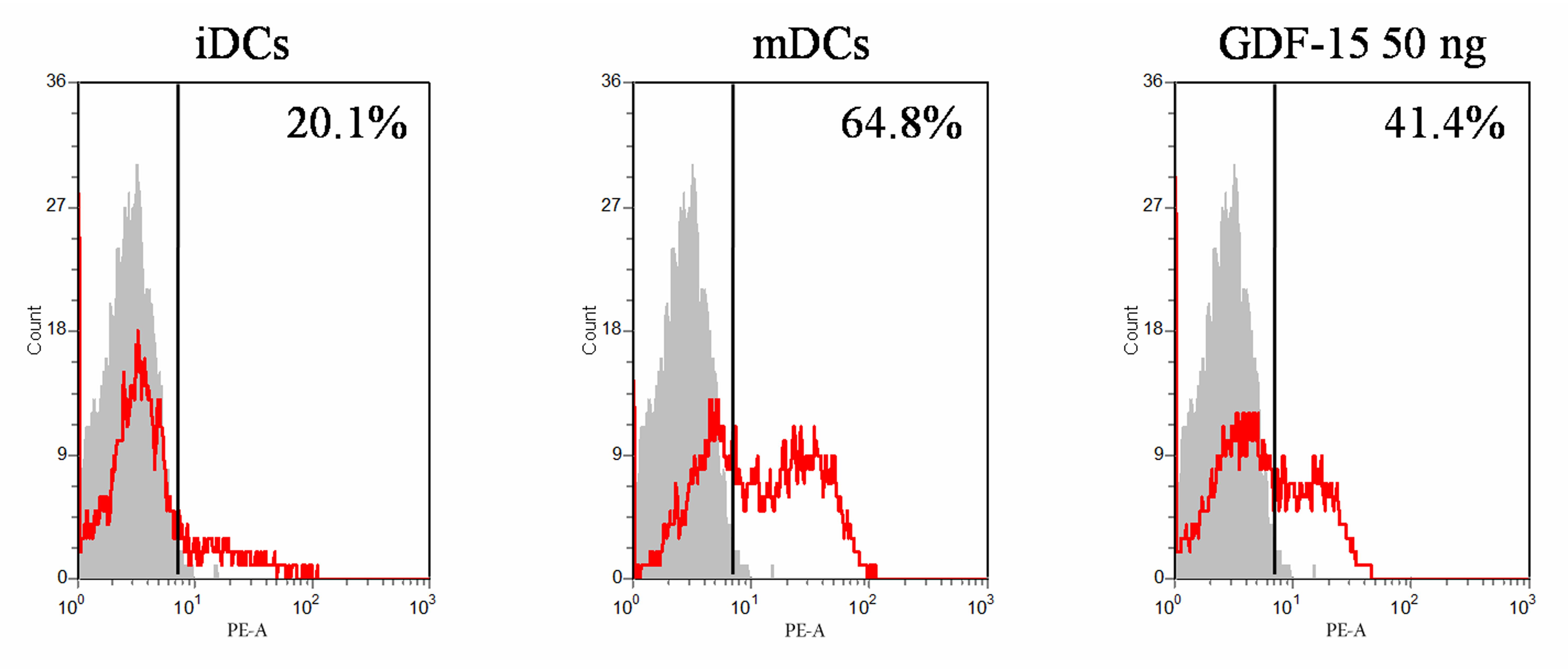

Supplement: Figure S5 — Measurement of CCR7 on murine DCs. DCs were harvested on day 8 for CCR7 analysis. Flow cytometry were performed to detect CCR7 expression. CCR7 expression on iDCs, mDCs and GDF-15 (50 ng/mL) treated DCs were 21.4±2.6%, 60.3±5.2% and 43.9±3.1%, respectively. Mean ± SD, n = 3. The experiments were conducted in triplicate. (TIF) [file pone.0078618.s005.tif]
